# The Effect of a Competitive Futsal Match on T Lymphocyte Surface Receptor Signaling and Functions

**DOI:** 10.3389/fphys.2018.00202

**Published:** 2018-03-15

**Authors:** Maria F. Cury-Boaventura, Renata Gorjão, Nivaldo R. de Moura, Vinicius C. Santos, José R. Bortolon, Gilson M. Murata, Leandro da Silva Borges, César M. Momesso, Alexandre Dermargos, Tania C. Pithon-Curi, Elaine Hatanaka

**Affiliations:** ^1^Institute of Physical Activity and Sport Sciences, Cruzeiro do Sul University, São Paulo, Brazil; ^2^Universidade Paulista, São Paulo, Brazil

**Keywords:** indoor soccer, inflammation, leukocytes, acquired immunity, cytokines

## Abstract

In this study, the lymphocyte activation status (surface expression of CD95, CD28, CD25, and CTLA-4), lymphocyte number, lymphocyte subpopulations, lymphocyte necrosis and/or apoptosis, and lymphocyte release of reactive oxygen species (ROS) were investigated in blood samples from 16 futsal athletes before and immediately following a competitive match. Lymphocytes were isolated from the blood samples, and the cellular parameters were assessed by flow cytometry. The futsal match induced lymphocytosis and lymphocyte apoptosis, as indicated by phosphatidylserine externalization, CD95 expression, and DNA fragmentation. Additionally, the competitive match induced the necrotic death of lymphocytes. No differences in the percentage of CD4+ and CD8+ T cells or in the T-helper/suppressor profile between before and immediately after the match were observed. Additionally, after the futsal match, the CD95 and CD28 expression levels were decreased, and the lymphocytes spontaneously released higher levels of ROS. Regardless of the origin, the situation-specific knowledge of lymphocyte behavior obtained herein may facilitate the design of strategies to control the processes that result in infection and tissue injury and that subsequently decrease athletic performance.

## Introduction

Futsal is a version of soccer that is generally played indoors on a pitch with a hard surface that is smaller than a soccer field. The game is played between two teams of five players each. The sport is characterized by aerobic and anaerobic metabolic demands, and the competitive futsal season includes weekly microcycles of training, tapering, competition, and recovery (Garcia-Tabar et al., 2015; Charlot et al., 2016; Sarmento et al., 2016). During a match, players may suffer injuries due to muscle fatigue and contact or collision among players, resulting in inflammation (de Moura et al., 2012, 2013; Beato et al., 2016). Specific knowledge regarding subclinical systemic inflammation and leukocyte function in athletes after a match may facilitate the design of strategies to control the inflammatory process, avoiding infection, and increasing athletic performance.

Lymphocytes are activated by the innate immune system, leading to the adaptive immune response. During these processes, naive T lymphocytes differentiate into specific effector cell subsets, resulting in a response to infection, inflammation, and physical exercise (Nielsen, 2003). Intense aerobic exercise at 80% maximal oxygen uptake induces lymphocytosis, which is largely mediated by adrenergic mechanisms (Nieman et al., 1994). Lymphocyte numbers return to normal levels within approximately 15 min and fall below normal levels within 1 or 2 h of intense exercise (Turner et al., 2010). This latter process reflects the extravasation of cells and is likely part of immune surveillance. Some authors have hypothesized that exercise might help to remove the excess accumulation of virus-specific T cells in the tissues via apoptosis (Simpson, 2011), but this hypothesis has yet to be proven experimentally. In response to pathogens, T helper cells (CD3+/CD4+) produce cytokines that impact on immune response, whereas cytotoxic T cells (CD3+/CD8+) produce chemicals that induce the death of infected cells. After these cells are recruited, a regulatory process is required to avoid excessive or uncontrolled immune activation (Burger and Dayer, 2002).

Moderate- and high-intensity exercise affects the Th1/Th2 balance. Studies have demonstrated that intense exercise suppresses the distribution and function of Th1 lymphocytes and that this effect is associated with the inhibition of the production of IL-12, an important cytokine related to Th1 induction, enhancing the capacity of monocytes to induce the synthesis of IL-4 by CD4+ lymphocytes (Hall et al., 2011). Acute exercise induces lymphocyte apoptosis via a pathway mediated by an external receptor (Fas-dependent signaling pathways) or mitochondrial mechanisms (via redox-sensitive pathways). Among the mechanisms of apoptosis in lymphocytes, TNF-α is associated with the initiation of the signaling cascade for cell death as well as stimulation for expression of CD95 on the membrane surface. The binding of CD95 present on cell surface to Fas ligand (Fas L) is a process that initiate cell apoptosis leading to an arrest of lymphocyte activation process (Blotta et al., 1997; Steensberg et al., 2001; Tuan et al., 2008; Krüger et al., 2009; Navalta et al., 2010; Walsh et al., 2011).

Playing futsal may alter lymphocyte numbers, death and activation status. During a futsal match, lymphocytes may be exposed to stress signals, including augmented levels of stress hormones, inflammatory cytokines, and ROS, which elicit changes at the molecular level that leave lymphocytes more susceptible to alterations. A comprehensive understanding of lymphocyte dysfunction and death may facilitate the design of strategies to control the processes that can result in infection and decreased athletic performance (Mars et al., 1998; Steensberg et al., 2001; Nielsen, 2003). In the present study, we determined the lymphocyte number and death (the proportion of cells without signs of necrosis and/or apoptosis, surface expression of CD95, release of reactive oxygen species, ROS), the percentage of viable CD4 and CD8 lymphocytes and the lymphocyte activation status (surface expression of CD28, CD25, and CTLA-4) in futsal athletes before and immediately after a competitive match.

## Materials and methods

### Subjects

Male volunteers (16 in total) participated in the study with the approval of the Ethics Committee of Cruzeiro do Sul University (protocol number 178/2008). The athletes signed an informed consent form to submit to the procedures performed in this study. The participants exhibited the following characteristics (mean ± SD): age 26.4 ± 3.2 years old, body mass 70.2 ± 6.9 kg, height 172.8 ± 5.7 cm, body fat 12.0 ± 2.3%, VO_2peak_ 59.7 ± 5.1 mL.kg^−1^.min^−1^, and sports experience as professional 4.4 ± 0.9 years. The professional athletes were futsal practitioners for at least 10 years and trained futsal 5 times per week 4 h per training period. All participants played intense exercise for approximately the same amount of time (5 min playing followed by 5 min of recovery, for a total of 10 min in each period) until they completed 2 equal periods (20 min in total). Subjects with a history of infection, viruses, chronic lesions, diabetes, rheumatoid arthritis, hormonal dysfunction, lupus, or other inflammatory or hematological diseases (such as hemoglobinopathies) and those who were taking medication were excluded from the study (de Moura et al., 2012, 2013).

### Sample collection

Venous blood samples (20 mL) were collected before and immediately after the futsal match. The site of blood collection was one of the 3 main veins of the antecubital fossa (the cephalic, basilic, or median cubital veins). The choice of vein depended on the identification of the optimal site, which involved both visual and tactile exploration (Monda et al., 2014). The blood was collected in two collection tubes; the first contained heparin and was used for plasma collection and cell separation, and the second was a gel dry tube used for serum collection.

The samples were collected in the field 1 h before and immediately after the match. After the sample collection, the serum was stored at ambient temperature (25–30°C), and the plasma was stored on ice. The experiments were performed within 1 h of the venepuncture, which was the time required to transport the samples from the field to the laboratory. In the laboratory, the blood was centrifuged (400 × g for 10 min), and the serum and plasma were separated from the cell components. Lymphocytes were immediately isolated, and the cellular functions were tested.

### Separation and isolation of blood lymphocytes

Lymphocytes (>99%) were isolated from the peripheral blood under endotoxin-free conditions using Histopaque® 1077 (Sigma Chemical Co., St. Louis, MO, USA) according to the manufacturer's instructions. The peripheral blood mononuclear cells (PBMCs, a mixture of monocytes and lymphocytes) were maintained in RPMI-1640 medium to allow the monocytes to adhere to the plates and to obtain a pure lymphocyte preparation (approximately 99% lymphocytes). The isolated lymphocytes were counted in a Neubauer chamber under an optical microscope (Nikon, Melville, NY).

### Cell death assays

PI is a popular red-fluorescent nuclear and chromosome counterstain. PI is an intercalating DNA agent that can be used to stain cells. Because PI is not able to permeate live cells, it is also commonly used to detect dead cells in a population. PI can be used to differentiate necrotic, apoptotic, and normal cells (Nicoletti et al., 1991).

### Cell viability assay (proportion of necrotic cells)

Lymphocyte (1.0 × 10^6^ cells/mL) viability was assessed using a FACSCalibur flow cytometer (Becton Dickinson Systems, CA, USA). The percentage of viable cells in each sample was determined based on PI staining (50 μg/mL). PI fluorescence was measured using the FL2 channel (orange-red fluorescence = 585/42 nm), and 10,000 events were analyzed per sample (Nicoletti et al., 1991).

### Proportion of cells with DNA fragmentation

Briefly, the lymphocytes (1.0 × 10^6^ cells/mL) were centrifuged at 1,000 × *g* for 15 min at 4°C. The resulting pellets were carefully resuspended in a hypotonic solution (300 μL) containing 50 μg/mL PI, 0.1% sodium citrate, and 0.1% triton X-100. Next, the cells were incubated for 30 min at 4°C. After staining the DNA with PI, DNA fragmentation was analyzed by flow cytometry. The presence of detergent in the solution permeabilized the cells, and the cells promptly incorporated the dye into their DNA. The PI fluorescence was measured using the FL2 channel (orange-red fluorescence = 585/42 nm), and 10,000 events were analyzed per sample. The flow cytometric data obtained using FACS and PI exhibit an excellent correlation with the results of DNA fragmentation that were obtained with both electrophoretic and colorimetric methods. The advantages of using flow cytometry are that the method is rapid, simple and reproducible (Steensberg et al., 2001).

### Annexin V staining of apoptotic cells

The annexin V-FITC method can be used to detect apoptosis because after initiating apoptosis, cells translocate the membrane phosphatidylserine from the inner face of the plasma membrane to the cell surface. Once on the cell surface, phosphatidylserine is detected by staining with a fluorescent conjugate of annexin V. Phosphatidylserine has a high affinity for annexin V. Lymphocytes (1.0 × 10^6^ cells/mL) were harvested from culture plates and centrifuged at 200 × g for 10 min at 4°C. The translocation of phosphatidylserine residues from the inner to the outer leaflet of the plasma membrane was assessed by their reaction with Annexin V-FITC (Clontech Laboratories, Inc., Palo Alto, CA, USA) and used as a measurement of apoptosis after analysis on a FACSCalibur flow cytometer (Becton-Dickinson, CA, USA) (Nicoletti et al., 1991).

### Flow cytometric measurement of reactive oxygen metabolites using hydroethidine

Hydroethidine is used for the flow cytometric measurement of intracellular ROS. Hydroethidine, a reduced derivative of ethidium bromide, penetrates cells, and exhibits weak fluorescence when excited. Hydroethidine is intracellularly oxidized by oxygen radicals and is converted into ethidium bromide, which tightly binds to DNA and exhibits strong red fluorescence. In our experiments, hydroethidine (1 μM) was added to the lymphocytes (1.0 × 10^6^ cells/mL) in incubation medium, and the cells were treated immediately with PMA (54 ng/mL). The release of ROS was monitored for 30 min. The samples were assayed in PBS supplemented with CaCl_2_ (1 mM), MgCl_2_ (1.5 mM), and glucose (10 mM) at 37°C in a final volume of 0.3 mL. The fluorescence was measured using the FL3 channel of a FACSCalibur flow cytometer (Becton Dickinson, CA, USA), and 10,000 events were analyzed per experiment (Hatanaka et al., 2006).

### CD95, CTLA-4, CD28, and CD25 membrane surface expression and CD4:CD8 ratio determination

The lymphocytes (1 × 10^6^ cells/mL) were resuspended in PBS and labeled with FITC-conjugated anti-CD95, anti-CD28, or anti-CTLA-4 antibody and APC-conjugated anti-CD25 antibody (1:50) (Becton Dickinson, San Juan, CA). The cell suspensions were incubated for 30 min at room temperature in the dark. Negative control cells were incubated with an isotype-matched non-reactive IgG1 antibody. Next, the cells were washed with PBS and analyzed using a FACSAria II flow cytometer (Becton Dickinson, San Juan, CA). In each experiment, 10,000 events were analyzed. The cells exhibiting FITC or APC fluorescence were evaluated using Diva software (Becton Dickinson), and the values were expressed as the mean fluorescence intensity. The percentages of CD4 and CD8 cells were determined after incubation with FITC-conjugated anti-CD4 and PE-conjugated anti-CD8 as previously described. The values were expressed as the percentage of total lymphocytes.

### Statistical analysis

The values are presented as the mean ± standard error of the 16 players. The statistical analysis initially consisted of parametric tests (*t*-test) demonstrating that the samples have a normal distribution. Then, one-way analysis of variance (ANOVA) was performed using the *post hoc* Student-Newman-Keuls multiple comparison test (INStat; GraphPad Software, San Diego, CA, USA). The significance level was set at *p* < 0.05.

## Results

**Figure 1:**
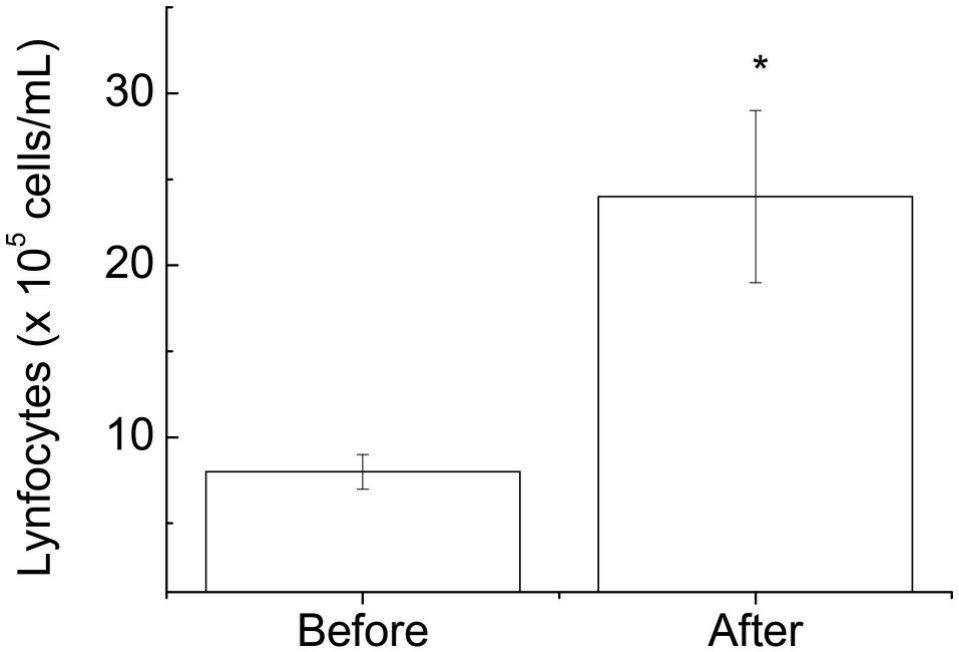
Blood lymphocyte number determined before and immediately after the futsal match. The values are presented as the mean ± standard error of 16 players. ^*^p < 0.01 for the comparison of the values between before and after the match.

**Figure 2:**
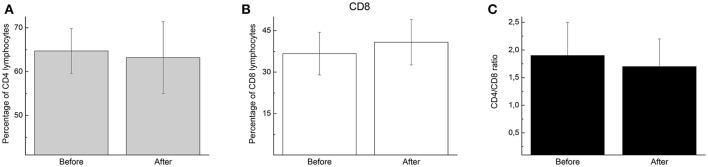
The percentage and ratio of CD4 and CD8 lymphocytes before and immediately after the futsal match. The values are presented as the mean ± standard error of 16 players. **(A)** The percentage of CD4 cells in the total lymphocyte samples. **(B)** The percentage of CD8 cells in the total lymphocyte samples. **(C)** The ratio of CD4 to CD8 cells in the total lymphocyte samples.

Lymphocytosis commonly occurs during and immediately after exercise, and the amount of lymphocytosis is proportional to the exercise intensity and duration. Player participation in the futsal match increased lymphocytosis (2.8-fold; *p* < 0.05; Figure 1) but did not alter the percentage of CD4 (helper) (Figure 2A) or CD8 (cytotoxic) T cells (Figure 2B) or the CD4:CD8 ratio (Figure 2C).

**Figure 3:**
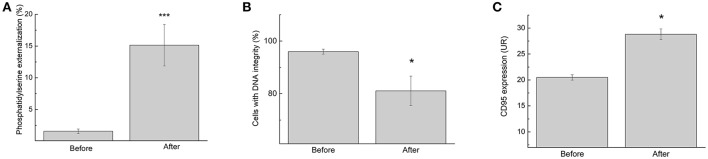
Lymphocyte apoptosis signaling determined before and immediately after the futsal match. **(A)** The percentage of cells with phosphatidylserine externalization. **(B)** The percentage of lymphocytes with DNA integrity. **(C)** Membrane surface expression of CD95. The values are presented as the mean ± standard error of 16 players. ^*^p < 0.05 and ^***^p < 0.001 for the comparison of the values between before and after the match.

**Figure 4:**
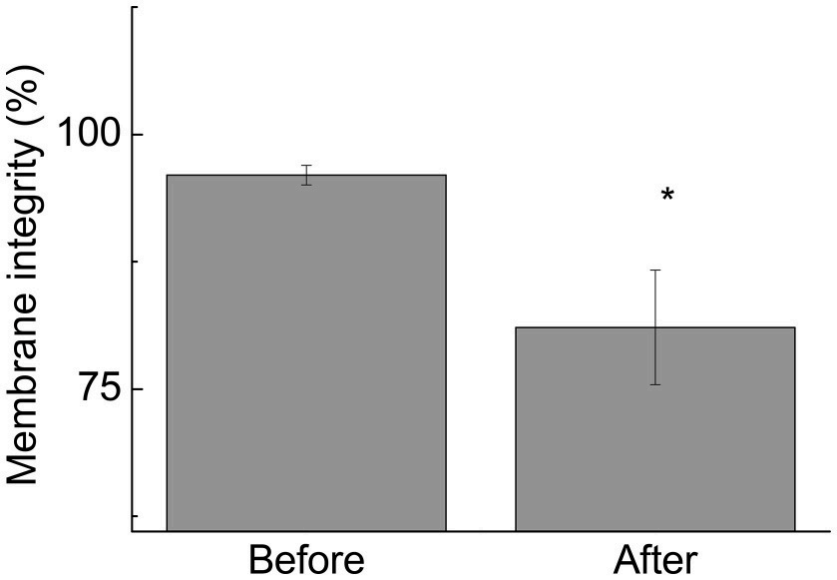
The percentage of lymphocytes exhibiting membrane integrity before and immediately after the futsal match. The values are presented as the mean ± standard error of 16 players. ^*^p < 0.05 for the comparison of the values between before and after the match.

Changes in lymphocyte number may lead to inappropriate activation of cells and even increased activation of cell death mechanisms, impairing an athlete's health. Therefore, the study was continued by measuring the proportion of cells with signs of necrosis and/or apoptosis. Although we observed an increase in the lymphocyte number, the futsal match induced lymphocyte death, as demonstrated by the increase in phosphatidylserine externalization (9.5-fold; *p* < 0.001; Figure 3A) and DNA fragmentation (1.1-fold; *p* < 0.05; Figure 3B). In addition, expression of CD95, a death receptor that mediates apoptosis and maintains immune homeostasis, was increased (1.5-fold; *p* < 0.05; Figure 3C). The competitive futsal match also decreased lymphocyte membrane integrity (1.4-fold; *p* < 0.05; Figure 4).

**Figure 5:**
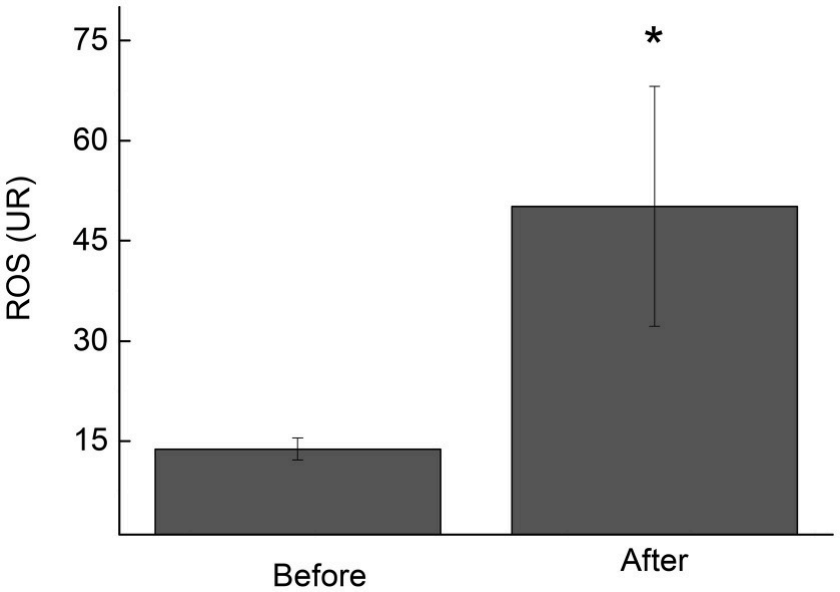
ROS release by lymphocytes before and immediately after the futsal match. The measurements were performed under basal conditions. The values represent the mean ± standard error of 16 players. ^*^p < 0.05 for the comparison of lymphocyte ROS production between before and after the match.

**Figure 6:**
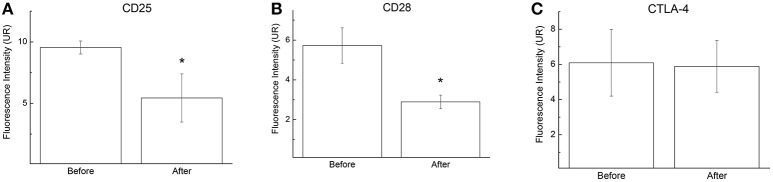
CD25 **(A)**, CD28 **(B)**, and CTLA-4 **(C)** expression on lymphocytes before and immediately after the futsal match. The cells were pelleted and labeled with APC-conjugated anti-CD25 (1:50), FITC-conjugated anti-CD28, and FITC-conjugated anti-CTLA-4 antibodies. Cells were analyzed by flow cytometry. Negative control cells were incubated with a labeled non-reactive control antibody. The values are presented as the means ± standard error of 16 players. ^*^p < 0.05 for the comparisons between before and after the match.

During a futsal match, lymphocytes are exposed to pro-apoptotic signals that include increased levels of cytokines and ROS (de Moura et al., 2012). Our results demonstrated that the lymphocytes that were collected immediately after the futsal match spontaneously released higher levels of ROS when stimulated with phorbol 12-myristate 13-acetate (PMA) (3.6-fold increase; *p* < 0.05; Figure 5). Additionally, CD25 and CD28 membrane surface expression levels decreased 1.7-fold (*p* < 0.05; Figure 6A) and 1.9-fold (*p* < 0.05; Figure 6B), respectively, after the competitive match. No alterations in CTLA-4 expression were observed under the conditions studied (Figure 6C).

## Discussion

The performance of futsal players depends on the physical, technical, tactical, psychological, and clinical characteristics of the individual players. From a clinical perspective, the impairment on immune function in athletes after intense exercise is common (Gleeson et al., 2006). Despite the popularity and competitive status of futsal, few studies have examined the health of players by focusing on immune cells (de Moura et al., 2012, 2013; Moreira et al., 2013).

We demonstrated that participation in the match induced lymphocytosis in the participants. No alterations in the numbers of CD4 or CD8 cells or in the CD4:CD8 ratio were observed. The cause of lymphocytosis is related to circulating catecholamines and alterations in the pro- and/or anti-inflammatory cytokine balance in response to exercise (Nieman et al., 1994; Nielsen, 2003; Walsh et al., 2011).

Our findings also indicate that the futsal match induced lymphocyte death through apoptosis, as indicated by phosphatidylserine externalization, DNA fragmentation, and CD95 expression. CD95 is categorized in the TNF-α family of receptors and is closely related to apoptosis (Teague et al., 1997; Chatzidakis and Mamalaki, 2010). CD95 activation pathways play an important role in the regulation of lymphocyte activity by preventing excessive self-activation (Burger and Dayer, 2002; Krüger et al., 2009). Additionally, the single competitive match increased lymphocyte necrosis. These phenomena may be attributed to an increase in lymphocyte ROS production or other metabolic or immune alterations (Turner et al., 2011). Apoptosis is considered an active cell death process and is characterized by the activation of proteases, the auto-destruction of chromatin, nuclear condensation, cellular membrane blebbing, and the vesicularization of internal components (Nicoletti et al., 1991). Although it has been reported that intense exercise induces lymphocyte apoptosis, the mechanisms and importance of these events are unclear (Mooren et al., 1985; Mars et al., 1998; Wang and Huang, 2005; Simpson et al., 2007; Neubauer et al., 2008; Navalta et al., 2013).

A number of authors have argued that apoptosis during exercise (i) is a physiological mechanism responsible to eliminate activated or excessive cells that enhance during exercise; (ii) is a normal regulatory process that removes certain damaged cells without inducing a pronounced inflammatory response; (iii) is a physiological response to excessive oxidative stress; and (iv) upregulates TNF-α, which is an important signaling molecule in apoptosis (Mooren et al., 1985; Mars et al., 1998; Wang and Huang, 2005; Simpson et al., 2007; Neubauer et al., 2008; Navalta et al., 2013). In a previous study, we observed an increase in TNF-α production by non-stimulated neutrophils in players after a futsal match (de Moura et al., 2012).

IL-6, TNF-α, and ROS elicit changes at the molecular level, leaving lymphocytes more susceptible to both the intrinsic and extrinsic pathways of apoptosis. In the extrinsic pathway of apoptosis, the activation of death receptors, such as CD95, causes the recruitment and oligomerization of the adapter molecule FAS-associated protein with the death domain within the death-inducing signaling complex (Reinehr and Häussinger, 2007). Furthermore, during apoptosis, membrane asymmetry is lost, phosphatidylserine translocates to the external leaflet of the cell surface, and cells undergo DNA fragmentation. Although we demonstrated an increase in lymphocyte ROS release, no alterations in the antioxidant capacity of the plasma or in TBARS (thiobarbituric acid reactive substance) measurements (data not shown) were observed.

The decrease in cell viability may led to the loss of lymphocyte control, thus promoting the decreased expression of important surface receptors such as CD28 and CD25. The T cell receptor engagement and the CD28/CTLA-4 signaling pathways play critical roles in T cell activation and regulation. CD28 engagement results in T cell activation, differentiation, and survival, whereas CTLA-4 signals blocks IL-2 production, cell cycle progression, and T cell differentiation (Fisher et al., 1995; Chen and Flies, 2013; Podojil and Miller, 2013).

In general, a decrease in the expression of CD25 is accomplish with an increase in CTLA-4. However, no differences in CTLA-4 expression on T lymphocyte membranes were observed after futsal match. In addition, studies have demonstrated that CD25-positive cells are more associated to Fas-mediated apoptosis (Krüger et al., 2009; Koncz and Hueber, 2012; Li et al., 2013).

Consistent with these data, we recently reported that playing futsal induces inflammation, activates neutrophils, and reduces the efficiency of neutrophil activity against infection resulting from the exposure to pathogens immediately after playing a futsal match (de Moura et al., 2012). The impairment on neutrophil activity, the main effector cell in innate immunity, and on lymphocyte activity, the main effector cell on adaptive immune function, suggest an immunosuppression which may contribute to increase the susceptibility of elite athletes to virus and bacterial infections.

A comprehensive understanding of lymphocyte dysfunction and death may facilitate the design of strategies to control the processes that can result in infection and decrease athletic performance. However, it is important to note that the mechanisms underlying futsal-induced changes in lymphocytes responses remain unknown and lie outside the scope of the present study.

The clinical consequences of repeated intense exercise, developed in sports as futsal and soccer, are the immunosuppression (Krüger and Mooren, 2014). The immunosuppression and the increase on susceptibility of infections have been demonstrated after endurance training or competition, however, our study was the first to reported an impairment in immunity in futsal players suggesting also immunosuppression after intense futsal training. It is also important to mention that lymphopenia is the result of both lymphocyte migration as well as apoptosis, hence, their relative intensity might depend on the different exercise protocols applied (Krüger et al., 2009). Regarding the prescription of physical training protocols, studies suggest that the level of apoptosis are progressively performed with increasing exercise intensity and that there is a specific threshold that cannot be exceeded (Mooren et al., 1985; Navalta et al., 2010). Therefore, in future research, it would be of interest to expand the present study in order to establish the duration/intensity of exercise which can be tolerated by futsal players before an elevation in the percentage apoptotic lymphocytes.

## Conclusions

In conclusion, our data demonstrate that playing futsal induces a decrease in the expression of lymphocyte activation markers (CD25 and CD28) as well as an increase in lymphocyte necrosis and apoptosis. During a futsal match, lymphocytes are exposed to pro-apoptotic signals, including augmented levels of inflammatory cytokines and ROS, that elicit changes at the molecular level that leave lymphocytes more susceptible to both the intrinsic and extrinsic pathways for apoptosis.

## Author contributions

Conceptualization: MC-B, RG, AD, and EH; Investigation and data curation: MC-B, RG, NdM, VS, JB, GM, LB, and CM; Development or design of methodology: MC-B, RG, TP-C, and EH; Formal analysis: MC-B, and RG; Project administration and funding acquisition: EH; Writing original draft: LB, AD, and EH; Writing review and editing: MC-B, RG, TP-C, AD, and EH.

### Conflict of interest statement

The authors declare that the research was conducted in the absence of any commercial or financial relationships that could be construed as a potential conflict of interest.
